# Ictal index finger pointing and politician's fist as localizing clinical signs in a pediatric patient

**DOI:** 10.1002/epd2.20323

**Published:** 2024-12-06

**Authors:** Joshua Chang, Mohamed Taha, Douglas Nordli

**Affiliations:** ^1^ Department of Pediatric Neurology University of Chicago Chicago Illinois USA

**Keywords:** encephalitis (anti‐NMDA receptor), frontal lobe (left), temporal lobe (left), tonic seizure, syndrome: focal non‐idiopathic frontal (FLE), etiology: encephalitis (anti‐NMDA receptor), phenomenology: motor seizure (complex), localization: frontal lobe (left), temporal lobe (left)

## Abstract

Content available: Video.

We report a case of a 3‐year‐old girl who presented with distinct, repetitive episodes featuring highly localizing clinical signs. Each episode began approximately 1 min and 50 s after the onset of electrographic changes, with the patient's right arm extending and her index finger assuming a pointed position (Video 1). Throughout the episode, her eyes largely remained closed, and she was unresponsive to her surroundings, unable to speak.

**VIDEO 1:** Scalp EEG captures an electroclinical seizure. Clinical signs were delayed by 1 min and 50 s after electrographic changes were noted. For the sake of video length, only the portion including clinical signs are included. Semiology features right arm extension and the ictal right index finger pointing, pincer, and politician's fist. Rhythmic spike discharges are maximal in the left frontocentral region, with subsequent spread into the left temporal chain (sensitivity: 20 μV/mm, LF: 1 Hz, HFF: 70 Hz, notch filter: 60 Hz). Video content can be viewed at https://onlinelibrary.wiley.com/doi/10.1002/epd2.20323

After about 20 s, the index finger flexed as her hand adopted a pincer posture, followed by the “politician's fist,” along with circular movements resembling “drawing circles in the air.” Electroencephalography (EEG) captured several episodes, with rhythmic spikes peaking in the left frontocentral region (F3/C3) and evolving into the left temporal chain (Video 1 and Figure 1).

**FIGURE 1:**
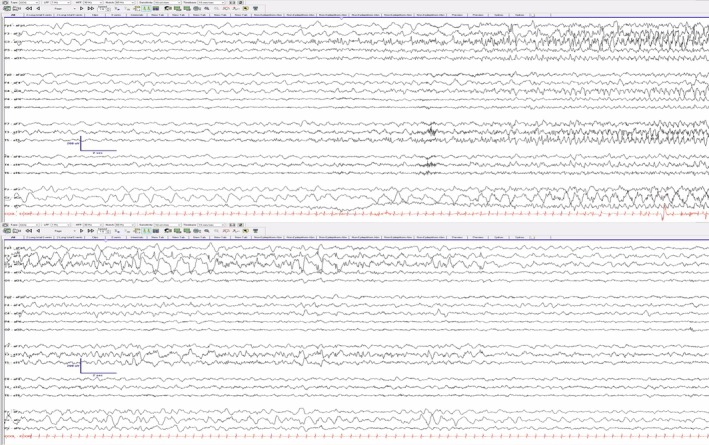
Ictal onset and offset pattern (Laplacian Montage) are included below to demonstrate the initial electrographic changes that preceded onset of clinical signs.

As the seizure resolved, her eyes briefly opened, and she momentarily attended to her mother as her right hand lowered. She then blinked before slipping back into stage 2 sleep, remaining postictal and asleep afterward. This was the only semiology that was noted in her case. Brain magnetic resonance imaging (MRI) was unremarkable; however, lumbar puncture revealed a positive titer for anti‐NMDA receptor (NMDAR) antibodies.

While ictal hand signs are well documented in adults, their localizing significance in children remains less understood. Previous studies suggest that the “ictal pointer, pincer, and politician's fist” signs often indicate seizure activity in the contralateral frontal and temporal regions.^1^, ^2^ Source localization software mapped the symptomatic zone to the left fronto‐temporal region (Figure 2), commonly associated with anti‐NMDAR encephalitis seizures.^3^ This case demonstrates how ictal signs can assist in accurately localizing focal seizures, further corroborated by diagnostic testing. The delayed clinical onset and downward propagation of the ictal field highlight that ictal semiology reflects seizure propagation rather than the precise seizure onset zone.

**FIGURE 2:**
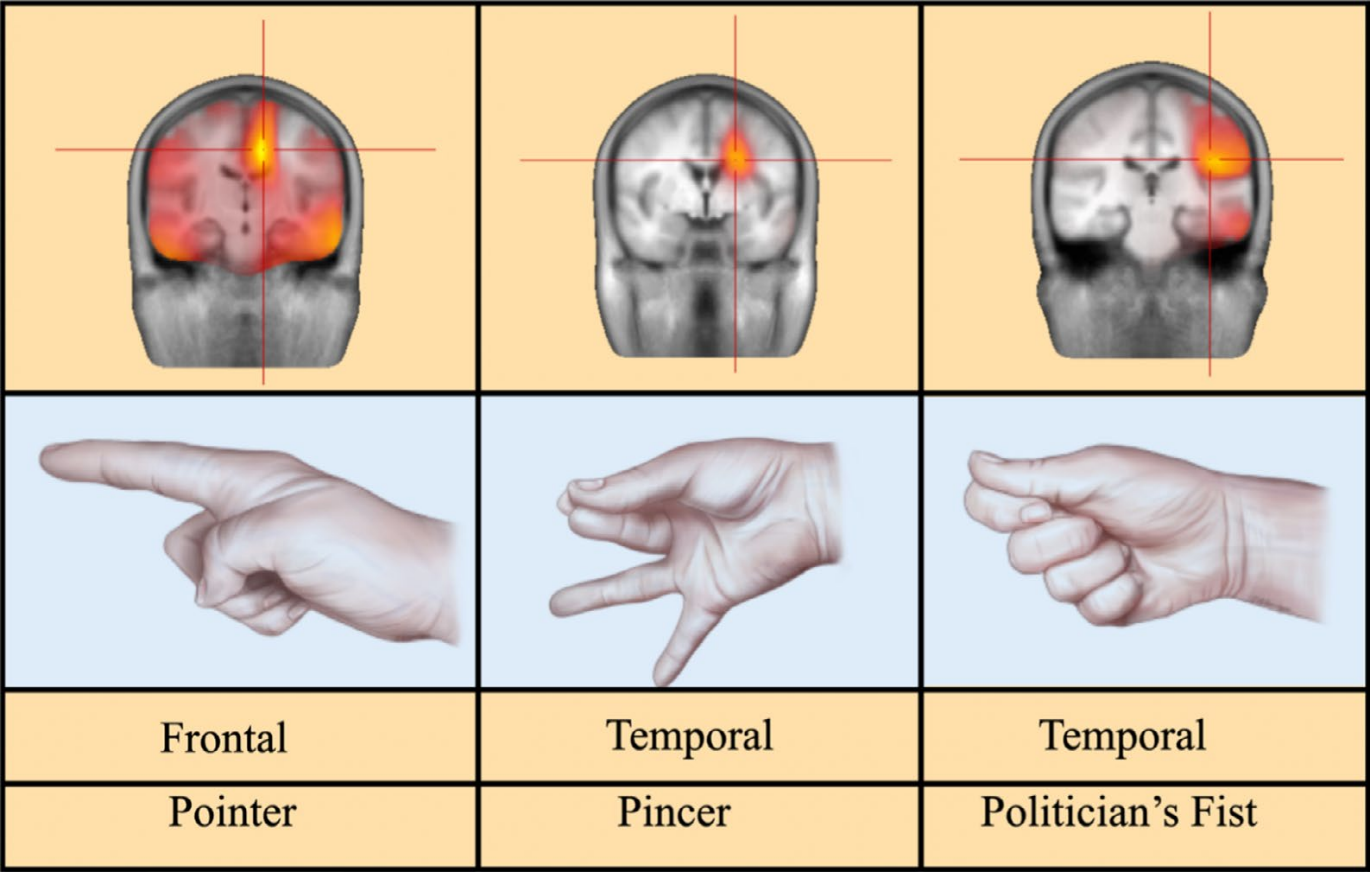
sLORETA current source density analysis was performed on the patient's EEG for each hand position change, displaying the downward propagation of the ictal field.

## CONSENT

Informed consent was obtained from the parents of the patient presented prior to beginning this work.

**Test yourself**

1. Ictal fist and pointer localizes most commonly to which area of the brain?
  - Ipsilateral fronto‐temporal region
  - Contralateral fronto‐temporal region
  - Ipsilateral parietal region
  - Contralateral occipital region
2. The “politician's fist” is an ictal hand sign that localizes most commonly to which area of the brain?
  - Contralateral temporal lobe
  - Contralateral frontal lobe
  - Ipsilateral frontal lobe
  - Ipsilateral parietal lobe
3. Seizures in anti‐NMDAR encephalitis have most commonly been associated with abnormal EEG findings in what area of the brain?
  - Fronto‐temporal region
  - Parietal region
  - Occipital region
  - No specific location

*Answers may be found in the* *Supporting information*.

## Supporting information


Data S1

Data S2
