# Supplementary material for: Ictal index finger pointing and politician's fist as localizing clinical signs in a pediatric patient
Source: Epileptic Disord. 2024 Dec 6;27(2):311–3. doi: 10.1002/epd2.20323 (PMC12065125; doi:10.1002/epd2.20323)
Supplement: Supplementary file 1 — Data S1 [file EPD2-27-311-s002.docx]

TEST YOURSELF

Answers:

1. B (contralateral fronto-temporal region). The finding of unilateral fist with index finger pointing has been shown to be relatively specific for ictal localization to the contralateral fronto-temporal region.

2. A (contralateral temporal lobe). The finding of a “politician’s fist” or a clenched fist without finger extension, has been shown to be relatively specific for ictal localization to the contralateral temporal region.

3. A (fronto-temporal region). Anti-NMDAR encephalitis related seizures seem to have a predilection for the fronto-temporal regions, as these have been the areas most implicated in focal slowing on EEG after seizures.
