# Supplementary material for: Ictal index finger pointing and politician's fist as localizing clinical signs in a pediatric patient
Source: Epileptic Disord. 2024 Dec 6;27(2):311–3. doi: 10.1002/epd2.20323 (PMC12065125; doi:10.1002/epd2.20323)
Supplement: Supplementary file 2 — Data S2 [file EPD2-27-311-s001.pptx]

## Slide 1
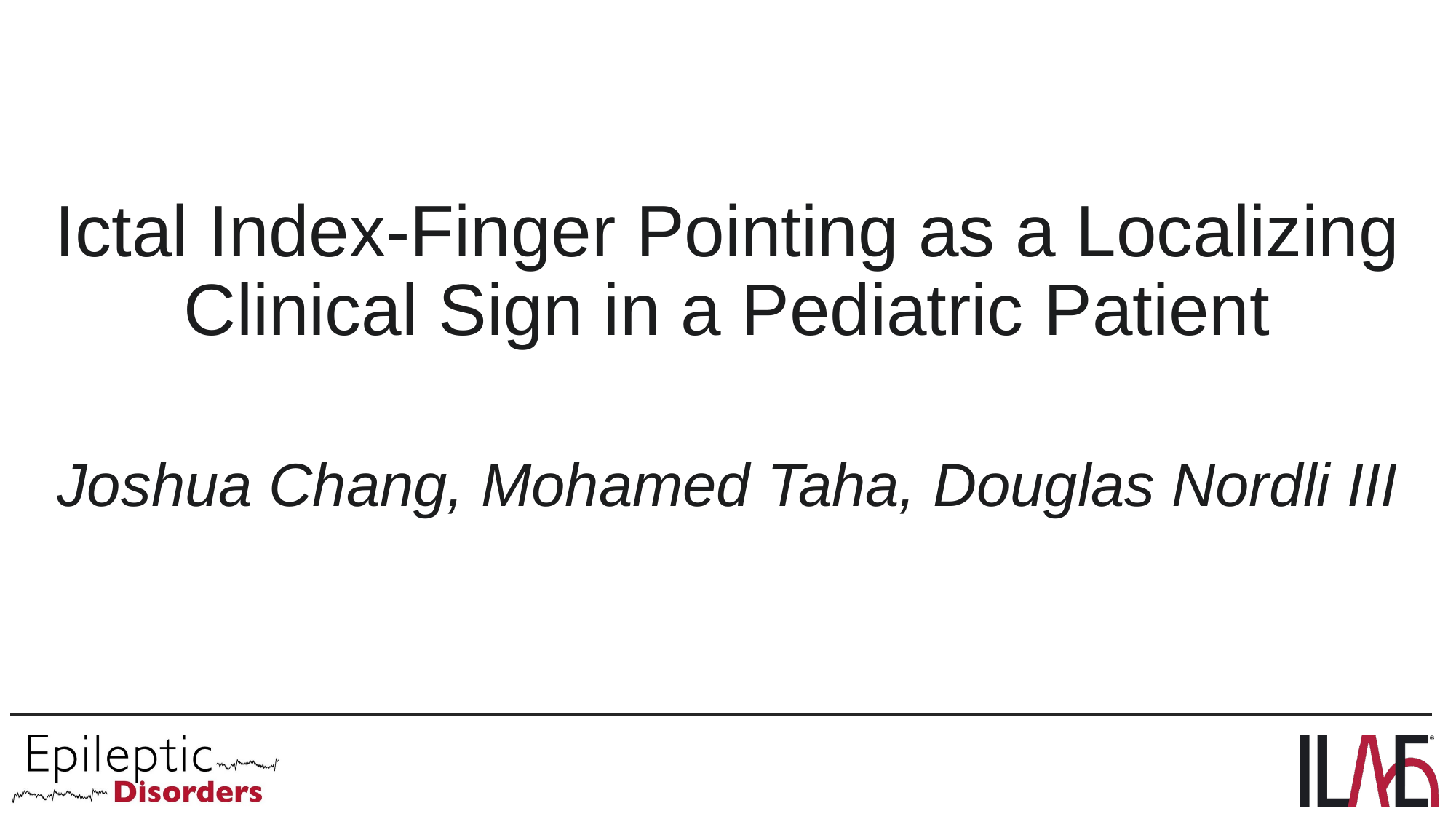

# Ictal Index-Finger Pointing as a Localizing Clinical Sign in a Pediatric Patient
Joshua Chang, Mohamed Taha, Douglas Nordli III

## Slide 2
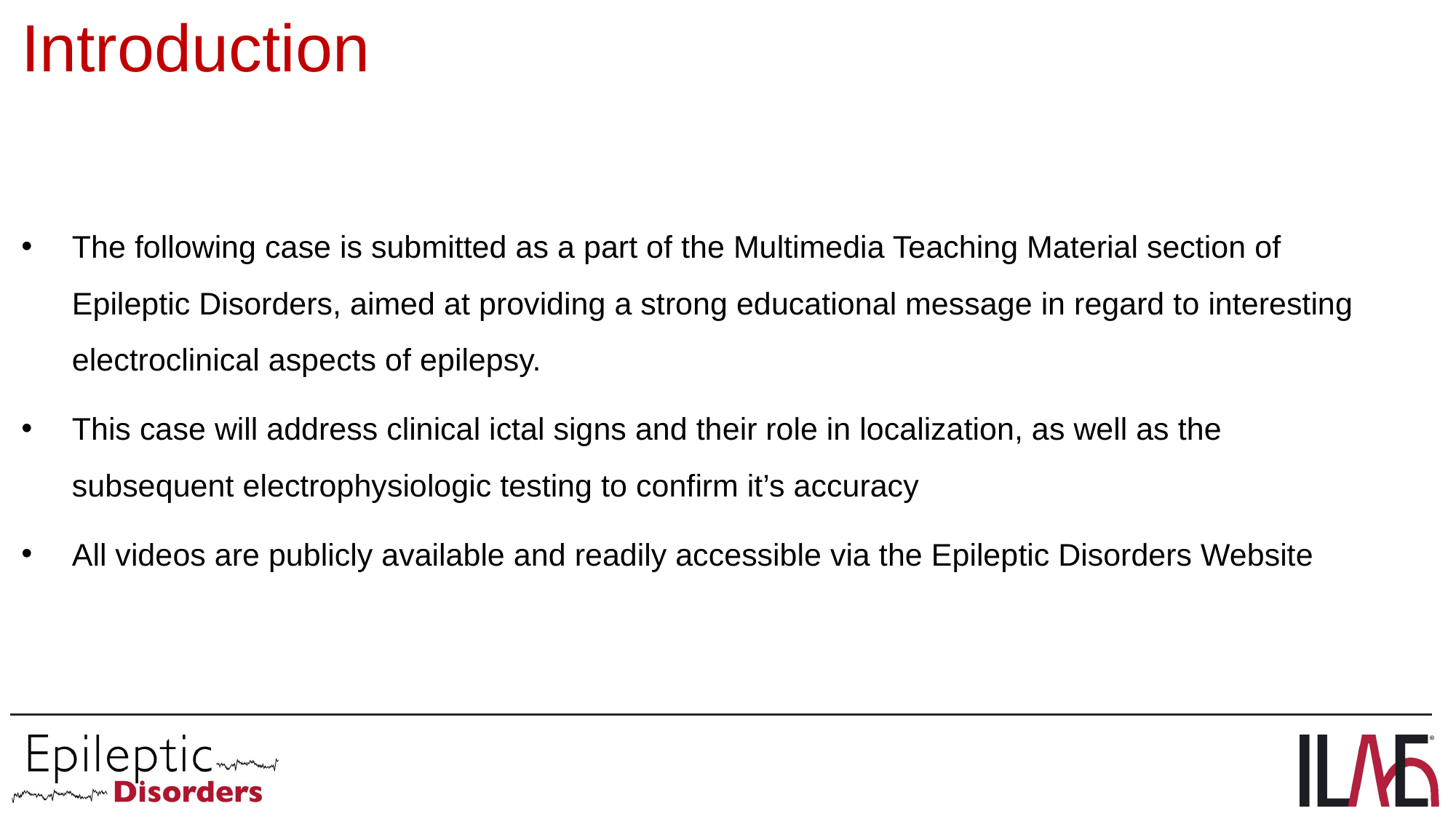

Introduction
The following case is submitted as a part of the Multimedia Teaching Material section of Epileptic Disorders, aimed at providing a strong educational message in regard to interesting electroclinical aspects of epilepsy.
This case will address clinical ictal signs and their role in localization, as well as the subsequent electrophysiologic testing to confirm it’s accuracy
All videos are publicly available and readily accessible via the Epileptic Disorders Website

## Slide 3
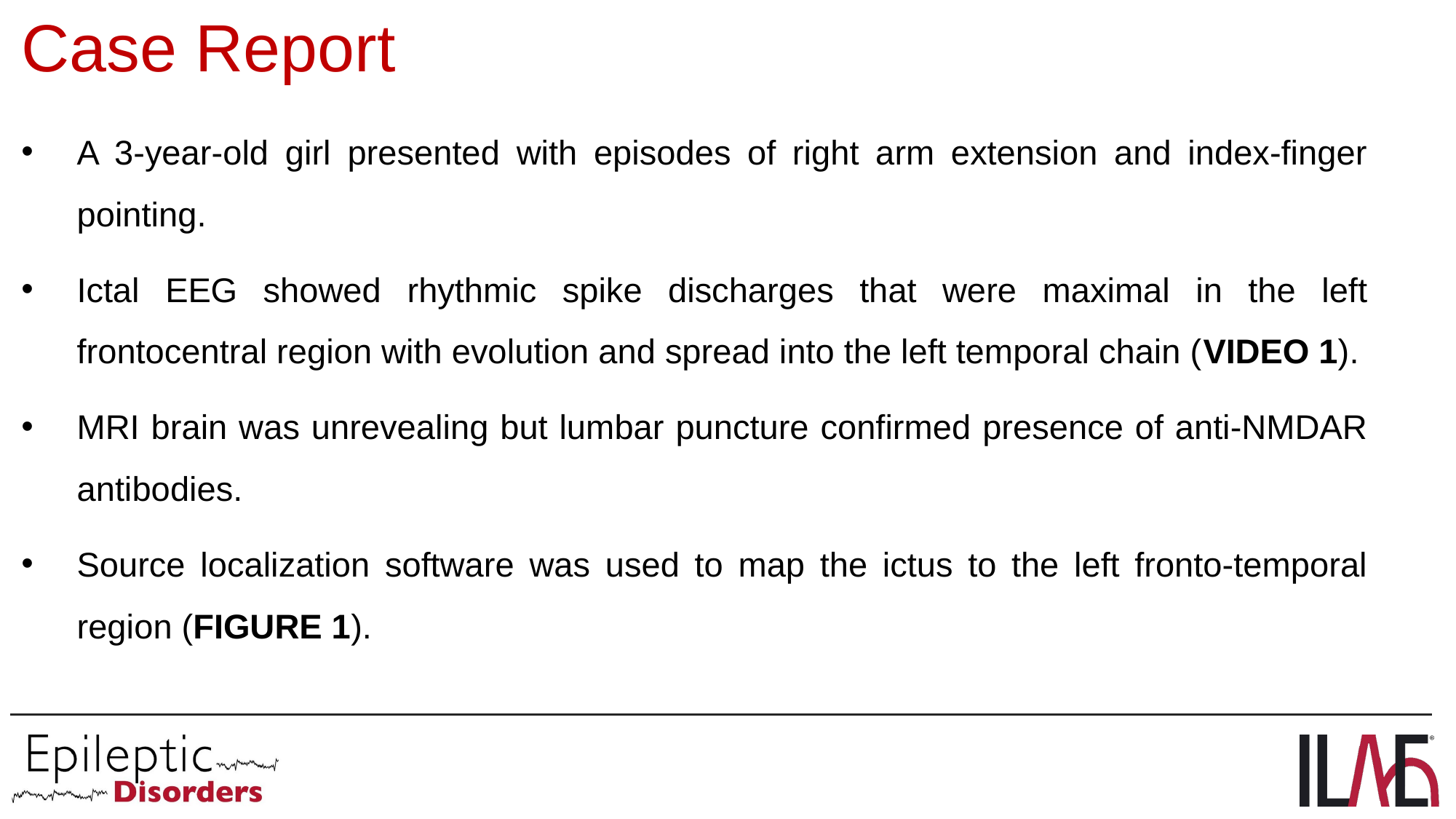

Case Report
A 3-year-old girl presented with episodes of right arm extension and index-finger pointing.
Ictal EEG showed rhythmic spike discharges that were maximal in the left frontocentral region with evolution and spread into the left temporal chain (VIDEO 1).
MRI brain was unrevealing but lumbar puncture confirmed presence of anti-NMDAR antibodies.
Source localization software was used to map the ictus to the left fronto-temporal region (FIGURE 1).

## Slide 4
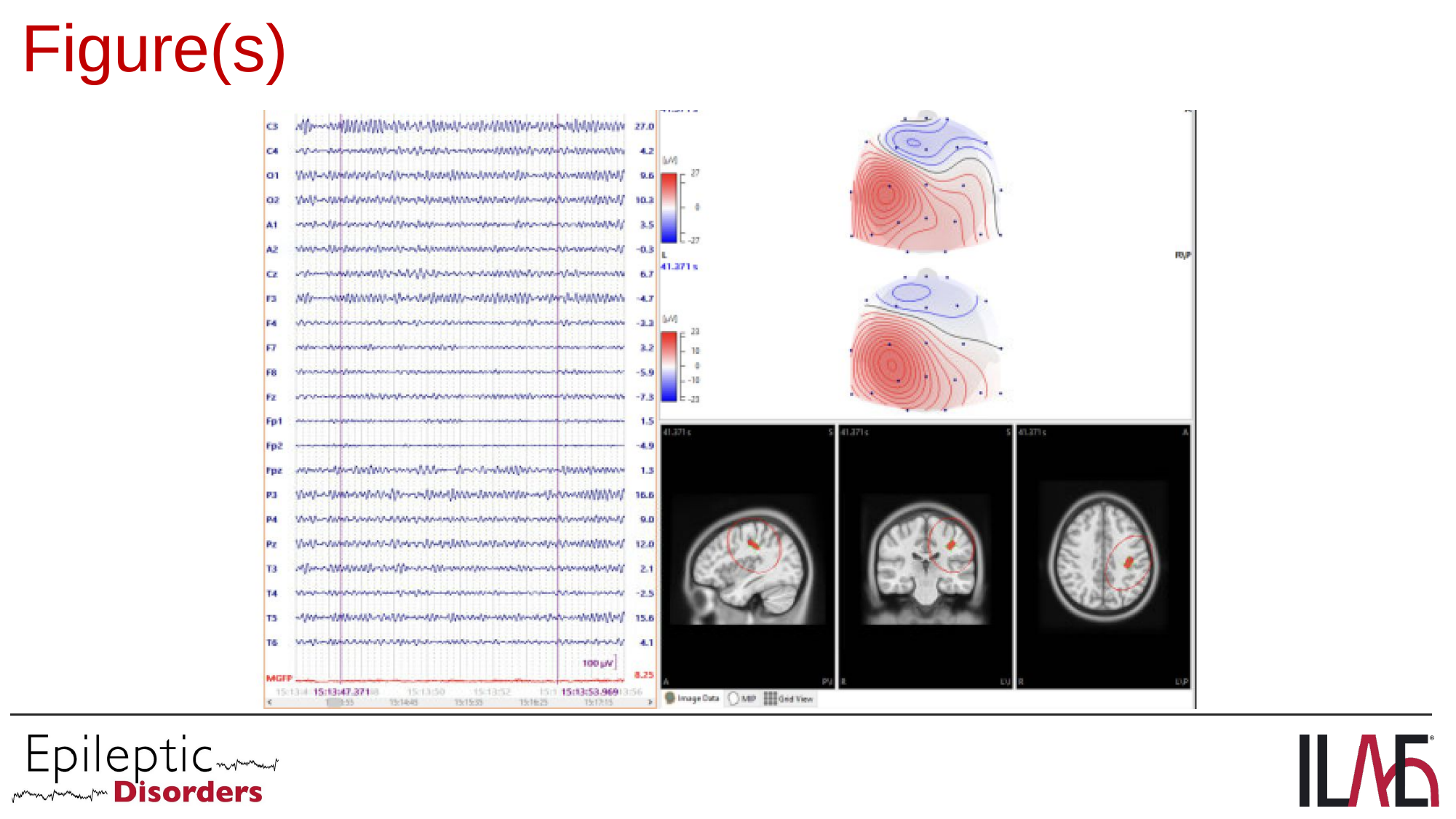

Figure(s)

## Slide 5
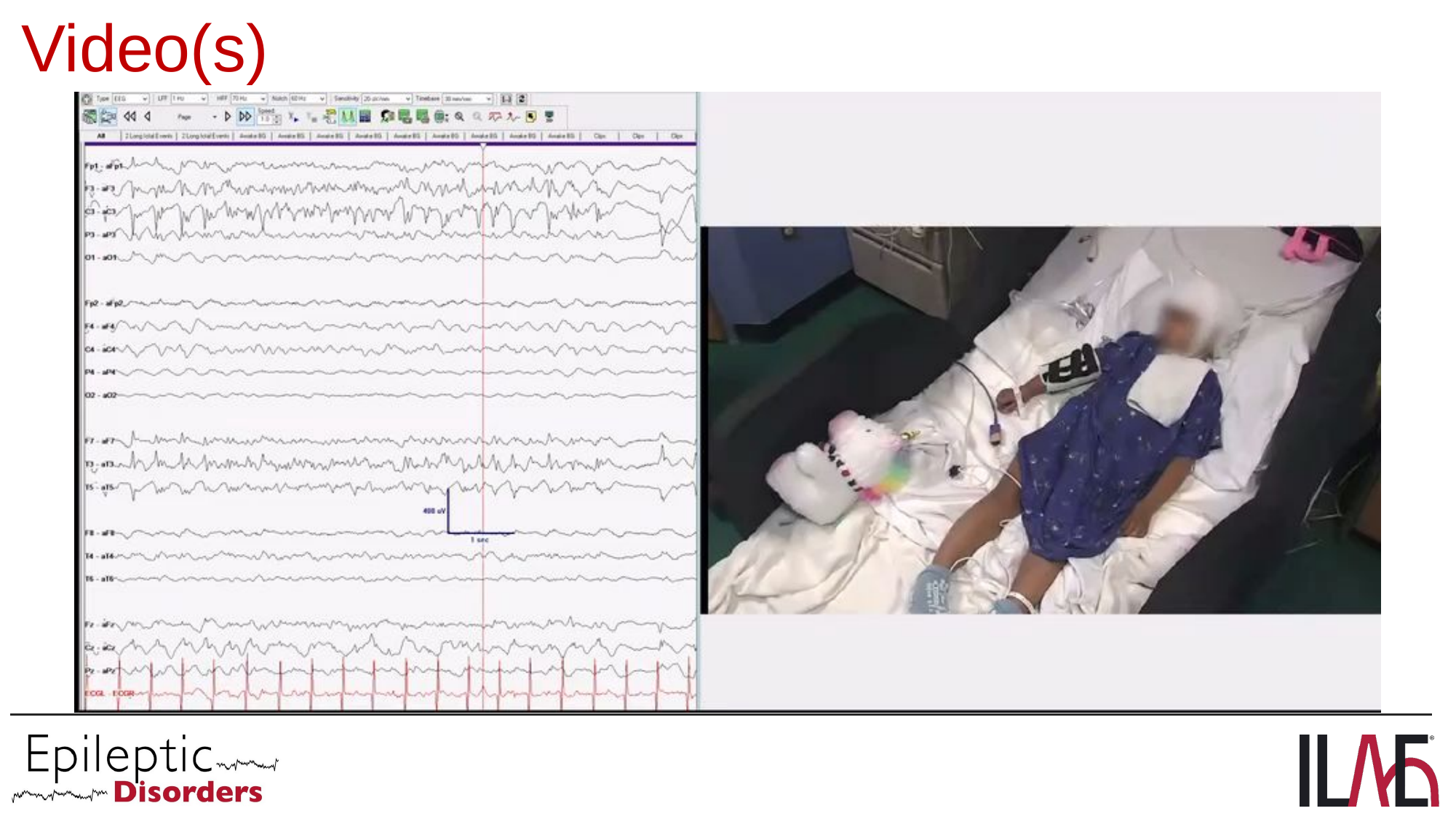

Video(s)

## Slide 6
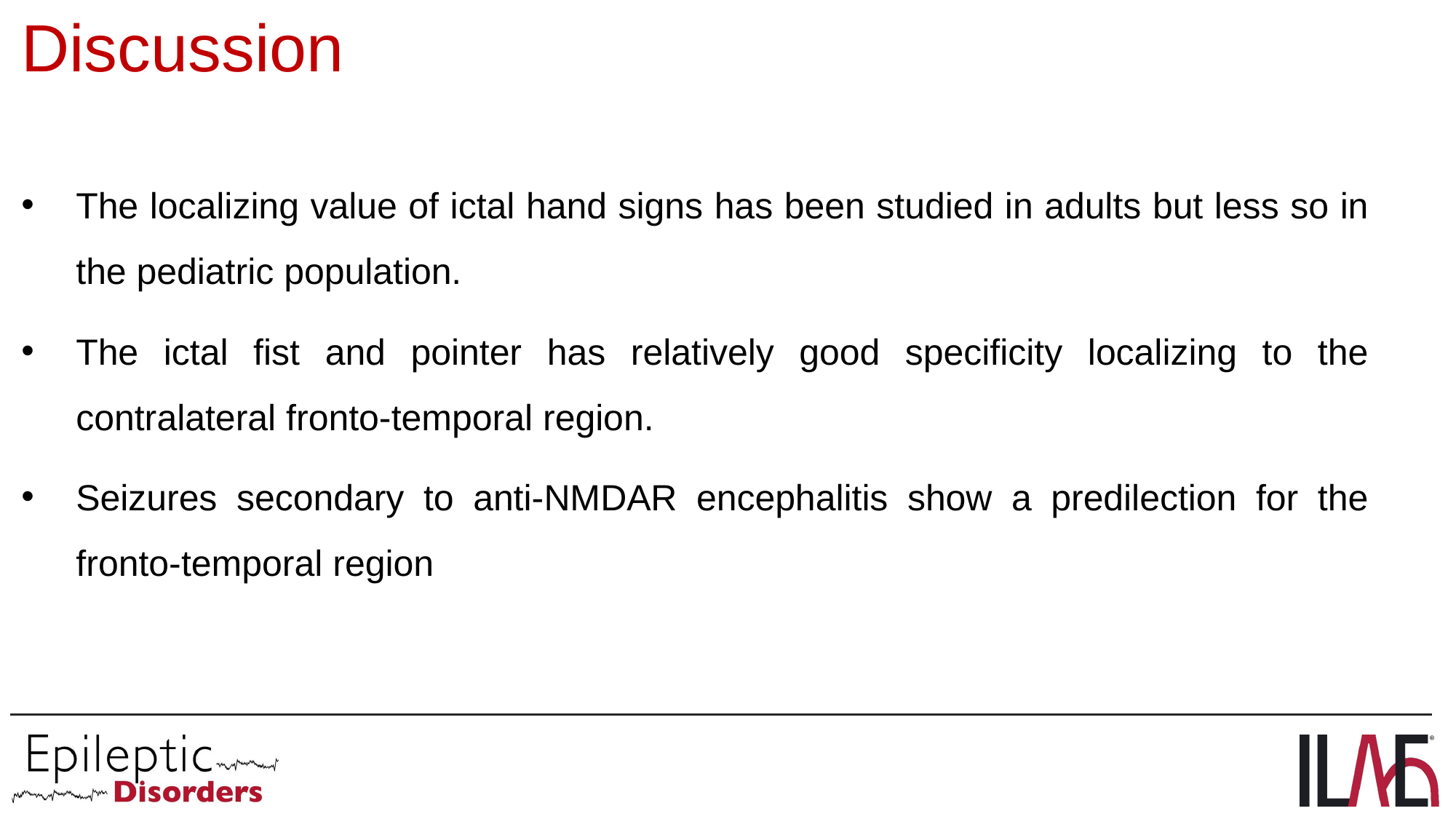

Discussion
The localizing value of ictal hand signs has been studied in adults but less so in the pediatric population.
The ictal fist and pointer has relatively good specificity localizing to the contralateral fronto-temporal region.
Seizures secondary to anti-NMDAR encephalitis show a predilection for the fronto-temporal region

## Slide 7
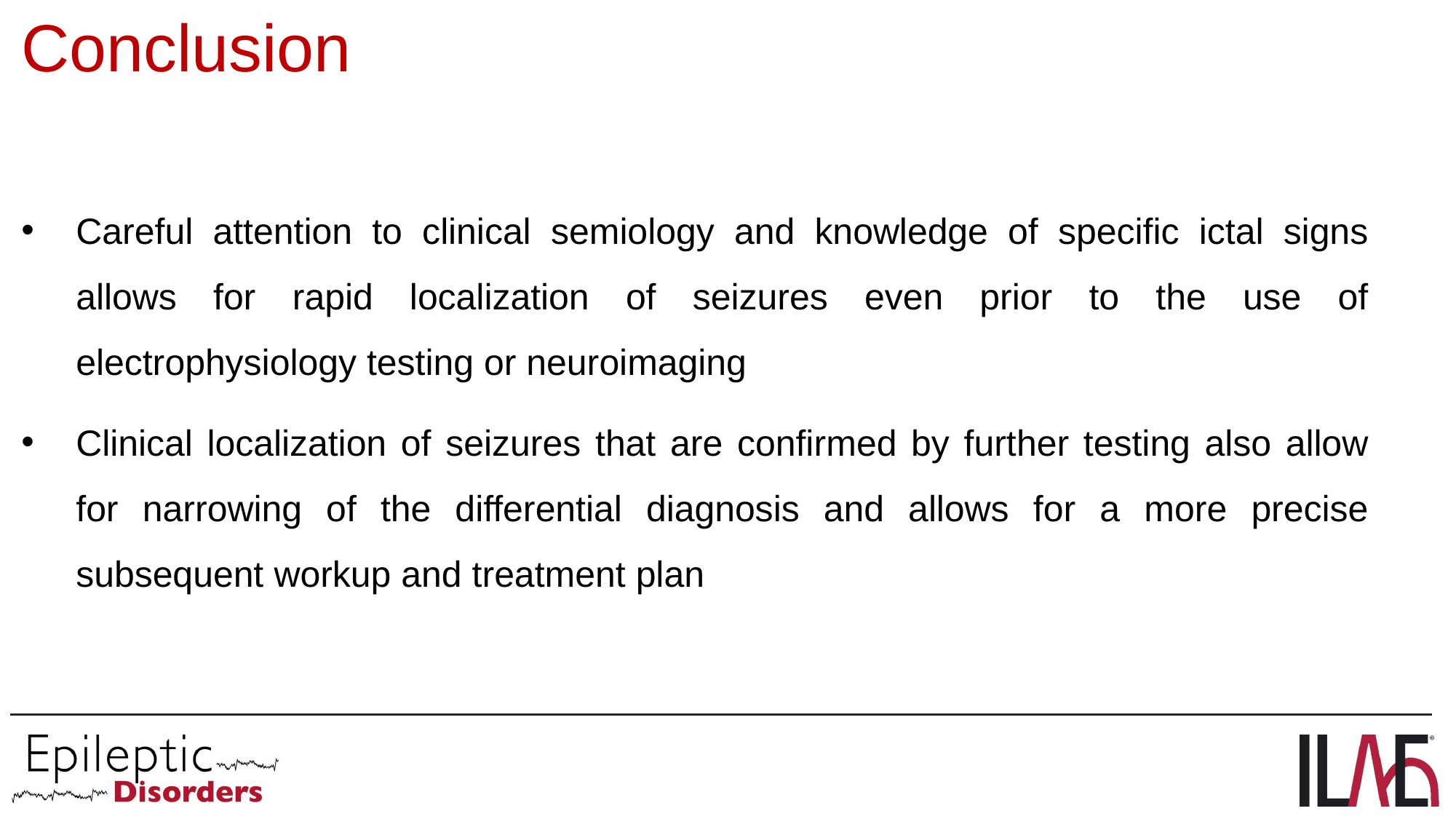

Conclusion
Careful attention to clinical semiology and knowledge of specific ictal signs allows for rapid localization of seizures even prior to the use of electrophysiology testing or neuroimaging
Clinical localization of seizures that are confirmed by further testing also allow for narrowing of the differential diagnosis and allows for a more precise subsequent workup and treatment plan
